# Intracortical Inhibition and Surround Inhibition in the Motor Cortex: A TMS-EEG Study

**DOI:** 10.3389/fnins.2019.00612

**Published:** 2019-06-12

**Authors:** Giorgio Leodori, Nivethida Thirugnanasambandam, Hannah Conn, Traian Popa, Alfredo Berardelli, Mark Hallett

**Affiliations:** ^1^IRCCS NEUROMED, Pozzilli, Italy; ^2^Human Motor Control Section, National Institute of Neurological Disorders and Stroke – National Institute of Health, Bethesda, MD, United States; ^3^Department of Human Neurosciences, Sapienza University of Rome, Rome, Italy

**Keywords:** electroencephalography, inhibition, motor cortex, SICI, TMS-EEG, transcranial magnetic stimulation

## Abstract

**Background:**

Short-latency intracortical inhibition (SICI) and motor surround inhibition (mSI) are cortical phenomena that have been investigated with transcranial magnetic stimulation (TMS). mSI is believed to be necessary for the execution of fine finger movements, SICI may participate in mSI genesis, and however, the mechanisms underlying both mSI and SICI are not entirely clear.

**Objective:**

We explored the cortical physiology of SICI and mSI in healthy subjects by TMS-evoked cortical potentials (TEPs).

**Methods:**

Single (sp) and paired-pulse (pp) TMS were delivered on the ADM muscle cortical hotspot while recording EEG and EMG. Three conditions were tested: spTMS and ppTMS at rest, and spTMS at the onset of an index finger movement. SICI and mSI were calculated on the ADM motor evoked potential (MEP) and two groups were defined based on the presence of mSI. Average TEPs were calculated for each condition and for five regions of interest.

**Results:**

At movement onset we observed a widespread reduction of the inhibitory late component N100 suggesting cortical facilitation associated with motor performance. At motor cortex level, SICI and mSI are associated with similar modulation of TEPs consisting in a reduction of P30 and an increase of N45 amplitude.

**Conclusion:**

Our findings suggest that SICI and mSI modulate cortical excitability with shared inhibitory mechanisms.

## Introduction

Short interval intracortical inhibition (SICI) can be investigated with transcranial magnetic stimulation (TMS) using paired-pulse paradigm where the motor evoked potential (MEP) produced by a test stimulus (TS) is reduced in amplitude if delivered 1–5 ms following a weak conditioning stimulus (CS) (Valls-Solé et al., 1992; Kujirai et al., 1993). It is postulated that SICI results from CS-induced recruitment of low-threshold cortical inhibitory interneurons responsible for inhibitory post-synaptic potentials (IPSPs) (Nakamura et al., 1997; Orth et al., 2003). Pharmacological studies suggest that SICI may be related to γ-aminobutyric acid type A receptor (GABA-Ar) activity (Ziemann et al., 1996; Di Lazzaro et al., 2006). Surround inhibition (SI) is a neurophysiological mechanism initially described in the sensory system by which the periphery of an activated neural network is inhibited while facilitating the center, thus increasing the spatial resolution (Blakemore et al., 1970; Angelucci et al., 2002). SI has been demonstrated in hand muscles with TMS delivered on the primary motor cortex (M1) (Sohn and Hallett, 2004). Because of motor SI (mSI), the MEP produced by a single pulse TMS (spTMS) in a surround muscle is reduced at the onset of a voluntary movement performed with a target/synergist muscle (Beck and Hallett, 2011). mSI should be helpful for the execution of fine finger movements and is thought to be the result of selective facilitation of muscles synergistic to the movement and inhibition of competitive ones (Hallett, 2003). Intracortical inhibitory mechanisms may therefore generate mSI. A previous study in healthy subjects found SICI in a surround muscle to be enhanced at movement onset (Stinear and Byblow, 2003) but a subsequent work did not replicate this result (Beck et al., 2008). The role of SICI in mSI is therefore unclear.

Recent technical progress has now enabled the concomitant use of TMS and EEG (Ilmoniemi and Kicić, 2010). The EEG recording of the TMS-evoked cortical potential (TEP) offers a new possibility to investigate cortical mechanisms in human beings (Ilmoniemi and Kicić, 2010; Hallett et al., 2017; Tremblay et al., 2019). TEPs over M1 are characterized by positive components peaking at 30, 60, and 180 ms (P30, P60, and P180), and negative components with latencies of 45 and 100 ms (N45, and N100) (Lioumis et al., 2009). While early components are thought to reflect the excitability of the stimulated cortex, the spatio-temporal distribution of the late components might reflect the connectivity of the stimulated area (Komssi and Kähkönen, 2006). By using the surface Laplacian operator (second spatial derivative of the voltage distribution), it is possible to extrapolate the Current Source Density (CSD) from scalp EEG (Kayser and Tenke, 2015). CSD represents the radial superficial dipole (i.e., the volume current flow out of the brain), so that signal amplitude reflects activity on the cortical surface where positive sign represents current flow from the cortex to the scalp, and negative sign represents to opposite direction (Nunez and Srinivasan, 2006). CSD estimates are rereference-free and have the advantage of critically reduce volume condition. Evoked potentials resulting from CSD transform have the same data domain of original potentials but are characterized by higher spatial and temporal resolution (Burle et al., 2015). Therefore, CSD estimates of TEPs are expected to provide accurate information on the cortical mechanisms underlying SICI and mSI.

Previous TMS-EEG studies have investigated TEP correlates of SICI providing conflicting results of either no effect (Paus et al., 2001), or significant modulation of different TEP components (Ferreri et al., 2011; Cash et al., 2017; Premoli et al., 2018). Also, while previous findings demonstrated N100 modulation during movement preparation, no studies have investigated the TEP correlates of movement performance and mSI (Nikulin et al., 2003; Bender et al., 2005; Kičić et al., 2008). Investigating the TEP correlates of SICI and mSI in healthy subjects may shed light on the inhibitory mechanisms controlling fine finger movements. In the present study on healthy subjects we investigated the effect of SICI, movement onset and mSI on the TEP produced by M1 stimulation. For this purpose, single and paired-pulse TMS were delivered during EEG recording with participants at rest and at the beginning of a finger movement.

## Materials and Methods

Twenty-four healthy volunteers (mean age 45.3 ± 10.2; M/F: 13/11), were enrolled after screening for eligibility. For eligibility, participants had to be 18–70 years old, right-handed (assessed through “Edinburg’s handedness inventory”) (Oldfield, 1971), able to give consent, able to carry out the study procedures, and abstain from alcohol for at least 48 h prior to study. Subjects were excluded for drug use in the previous 6 months, more than 14 alcoholic drinks/week for men and 7 alcoholic drinks/week for women, neurological abnormalities, history of brain tumor, stroke, head injury with loss of consciousness, seizure disorder, major psychiatric disorders, medication that would influence central nervous system function, and presence of metal in the cranial region (DBS, metal clips, cochlear implants, and fragments, etc.), pacemakers, hearing loss, and pregnancy. This study was carried out in accordance with the recommendations of Declaration of Helsinki with written informed consent from all subjects. The protocol was approved by the Neuroscience Institutional Review Board of the National Institutes of Health.

Two Magstim2002 stimulators connected through a Bistim unit (Magstim Company, United Kingdom) and to a 90 mm figure-of-eight coil were used to deliver monophasic spTMS and paired-pulse TMS (ppTMS). Single-pulse TMS was delivered in the Bistim mode by setting the intensity of one stimulator to 0% of the maximum stimulation output, and the intensity of the other stimulator at test stimulus intensity (see below). The coil was held tangential to the scalp at an angle to the mid-sagittal plane so as to induce a postero-anteriorly directed current perpendicular to the central sulcus. TMS was applied over left M1 at the spot that evoked the largest MEP in the contralateral abductor digiti minimi (ADM) muscle (“hotspot”). This position was stored in a neuro-navigation system and monitored during the experiment. We estimated the resting motor threshold (RMT) as the minimum intensity that evoked an MEP of 0.05 mV in 50% of trials using the adaptive threshold hunting procedure (Awiszus, 2003). MEP recruitment curve was recorded for the ADM by delivering spTMS at intensities ranging from 5 to 100% of the maximum stimulation output, with 5% step width, and 3 trials per intensity. Input/Output curve (IOC) was obtained by fitting a Boltzman equation-defined sigmoid curve on the stimulation intensity vs. MEP amplitude plot (Kukke et al., 2014). Using the IOC, we calculated the intensity that generated an MEP of 50% of the maximum amplitude (S50). The S50 was used as test stimulus (TS) intensity, while 80% of the RMT was used as conditioning stimulus (CS) intensity. SICI was investigated by delivering a TS 2 ms following a CS (Ilic et al., 2002). To study mSI, TS was delivered at the onset of a flexion movement of the index finger, identified as the moment when the electromyographic (EMG) activity recorded on the first dorsal interosseous muscle (FDI) exceeded 0.1 mV.

Neuronavigation (Brainsight, Rogue Research, Inc., United Kingdom) with an optical tracking system (“Polaris Vicra”, Nothern Digital Inc., Canada) was used for precise positioning of the coil. We digitized 5 reference points – nasion, nose tip, left, and right pre auricular, and left and right outer canthi of the eyes of each subject on to a reference MRI image and marked the hotspot of ADM on it. Using this hotspot as target, we monitored the position of the coil throughout the experiment session. The coil was always positioned over the empirically determined hotspot with an acceptable error of less than 2 mm.

The EMG activity was recorded through pairs of Ag/AgCl surface electrodes placed in a belly-tendon montage over the FDI (synergist muscle), and ADM (surround muscle). The EMG signal was amplified and filtered (20 Hz–1 kHz bandwidth) with a Nihon Kohden EMG machine (Nihon Kohden Corporation, Tokyo, Japan), and digitized at 5 kHz with a CED 1401 A/D laboratory interface (Cambridge Electronic Design, Cambridge, United Kingdom). Data were archived in a laboratory computer for on-line display and further off-line analysis (Signal version 6, Cambridge Electronic Design, Cambridge, United Kingdom). To ensure complete relaxation of target muscles during rest periods, we continuously monitored EMG activity and gave verbal feedback to participants. EEG was recorded from 32 channels using a TMS compatible system (NeurOne Tesla, Bittium, Finland). The scalp of each subject was prepared for EEG recording by cleaning with alcohol and subsequent application of abrasive and conductive gel. A TMS-compatible elastic cap (BrainCap, EASYCAP, Herrsching, Germany) with 64 electrodes mounted in a 10%-System layout was placed on the participants’ head. The EEG signal was digitized with a sampling frequency of 10 kHz from the following channels: Fp1, Fp2, AFz, F7, F3, Fz, F4, F8, FC5, FC1, FCz, FC2, FC6, T7, C3, Cz, C4, T8, TP9, CP5, CP1, CP2, CP6, TP10, P7, P3, Pz, P4, P8, O1, O2, and Iz. As reference for all the electrodes, a further channel was placed on POz and an electrode positioned on Fpz was used as ground. We made sure to maintain an impedance of less than 5 kΩ for each of the 32 recording electrodes and ground electrode.

During the experiment, the participants were seated comfortably on a chair, with the right forearm placed on a table with a cushion to allow complete relaxation. Subjects were asked to keep their eyes open, remain vigilant for the duration of the experiment, and wear earplugs to reduce the contamination of the EEG signal by acoustic stimuli. The experiment comprised of 3 randomized blocks with 100 trials each. In each trial, participants were asked to perform a brief (<1 s duration) flexion with the right index finger after a self-paced delay of about 3 s following an auditory stimulus (Kassavetis et al., 2012). The delay after the acoustic stimulus was included to reduce possible contamination by auditory evoked potentials. The participants were instructed not to move the remaining fingers and to relax in the interval between movements. In one block, spTMS was delivered at rest, i.e., 5 s after the execution of the movement (restTS). In a second block, ppTMS was delivered at rest according to the SICI paradigm (restPP). In a third measurement block, spTMS was delivered at the movement onset, i.e., the moment in which EMG activity in the FDI muscle reached the 0.1 mV threshold (MovTS). The order of the blocks was randomized for each subject to avoid possible bias introduced by the long experimental session and by plasticity phenomena induced by the repeated movement if any (Kassavetis et al., 2012; Belvisi et al., 2014).

### Data Analysis

**FIGURE 1:**
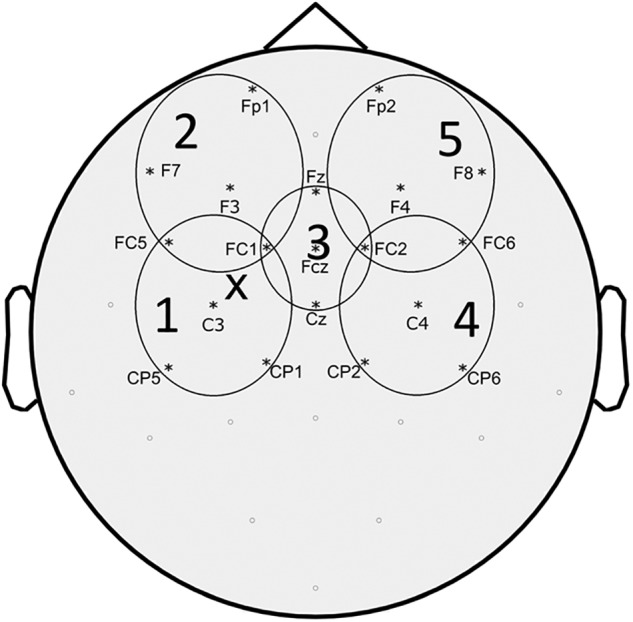
Topographic representation of the EEG electrodes included in each region of interest (ROI) considered for TEPs analysis. “X” represents approximate site of stimulation.

Preprocessing of EEG data was performed using the Fieldtrip, a MATLAB-based open source toolbox (Oostenveld et al., 2011) following a pipeline similar to what described in previous studies (Rogasch et al., 2014). The continuous EEG signal of each block was separated into epochs from -1000 ms to +1500 ms around the TMS pulse. Trials were inspected and those excessively contaminated by artifacts (eye movement artifact, blinking, movement, bad electrode contact, EMG, etc.) were eliminated. The TMS artifact (or artifacts in dual-stimulus trials) was removed by eliminating EEG data from 5 ms before to 5 ms after the TMS pulse. Since we were interested in characterizing cortical phenomena circumscribed at motor cortex level, we applied a Laplacian filter to our EEG signal to reduce volume conduction (Nunez and Srinivasan, 2006; Carvalhaes et al., 2009). Scalp surface Laplacian was computed using the “CSD,” a MATLAB-based open source toolbox based on the spherical splines method (Kayser and Tenke, 2006a,b; Kayser, 2009). Subsequently, we performed an independent component analysis for the elimination of the components related to eye movements and blinking, as well as decay artifact from the TMS pulse. The residual TMS-evoked muscle artifact was removed and replaced with Not-a-Numbers (NaNs) later substituted by cubic interpolation. Finally, EEG signal was filtered (1–50 Hz), down-sampled (1 kHz), and baseline corrected by subtracting the mean signal amplitude calculated between -500 and -100 ms before the TMS stimulus. After pre-processing, TEPs were obtained for each block by averaging across trials from 100 ms before to 300 ms after TMS. Since we were interested in the effect of movement onset on TEPs, we subtracted the averaged movement related potential (MRP) from the TEPs of the MovTS condition. The MRP was obtained from the restTS block by averaging 500 ms of EEG after movement onset (see above for definition of movement onset). We defined 5 predetermined regions of interest (ROI) based on previous studies (Cash et al., 2017) and calculated the average TEPs of the 5 EEG channels included in each ROI (ROI 1 – “left fronto-parietal”: FC5, FC1, C3, CP5, CP1; ROI2 – “left prefrontal” Fp1, F7, F3, FC5, FC1; ROI3 – “centro-medial”: Fz, FC1, FCz, FC2, Cz; ROI4 – “right fronto-parietal”: FC2, FC6, C4, CP2, CP6; ROI5 – “right prefrontal”: Fp2, F4, F8, FC2, FC6) (Figure 1). The 300 ms post-stimulus was divided into 5 time intervals of interest (TOI), corresponding to the 5 main TEP peaks: P30, N45, P60, N100, and P 180 (Lioumis et al., 2009). TOI were decided by observing participants’ grand average TEP in the centro-medial ROI (ROI3) at RestTS, and were kept consistent across conditions: P30: 20–37 ms, N45: 38–48 ms, P60: 48–65 ms, N100: 85–140 ms, P180: 150–230 ms. For each ROI and condition, TEP peak amplitudes were measured as the most positive/less negative value in TOI corresponding to positive components, and the most negative/less positive value in TOI corresponding to negative components. The average amplitude (peak-to-peak) of MEPs from ADM and FDI was calculated for each condition. SICI was calculated as the ratio between MEP amplitudes at restPP and restTS. The mSI was calculated from the ADM MEP as the ratio between MovTS and RestTS, and a cut-off value of 0.8 for this ratio was defined as a threshold for mSI presence. Participants were offline assigned either to the “mSI” group (ratio < = 0.8) or to the “NOmSI” group (ratio > 0.8). Since we were looking for correlates to mSI and from past studies it is known that only some subjects show mSI, this division of subjects was an *a priori* design of the experiment. We used a histogram graph to assess the distribution of our participants based on mSI. Finally, movement facilitation (mF) was calculated from FDI MEP as the ratio between MovTS and RestTS.

### Statistical Analysis

Paired *T*-test was used to compare ADM MEP between RestTS and RestPP (SICI), and between RestTS and MovtTS (mSI). To investigate movement-associated effect on FDI MEP, paired *T*-test was used to compare RestTS and MovtTS conditions. Unpaired *T*-test was used to investigate differences in ADM MEP between mSI and NOmSI groups at RestTS to exclude possible differences in baseline values. In order to investigate differences in the movement-associated facilitation on FDI MEP between mSI and NOmSI group, we performed a mixed-design two-way ANOVA with Condition (within-subject, levels: RestTS and MovtTS) and Group (between-subject, levels: mSI and NOmSI) as main factors.

To investigate the SICI-associated effect on TEPs, we carried out a three-way repeated measures ANOVA with factors ROI (within-subject, levels: 1, 2, 3, 4, and 5), Condition (within-subject, levels: restTS vs. restPP), and Component (within-subject, levels: P30, N45, P60, N100, and P180).

To investigate the effect of movement onset and mSI on TEPs amplitude, we performed a four-way mixed ANOVA with factors ROI (within-subject, levels: 1, 2, 3, 4, and 5), Condition (within-subject, levels: restTS vs. movtTS), Component (within-subject, levels: P30, N45, P60, N100, and P180) and Group (between-subject, levels: mSI and NOmSI).

Pairwise comparisons on significant main effects were performed and corrected for multiple comparisons with Bonferroni correction.

A value of *p* < 0.05 was considered significant. The sphericity in data distribution was verified by Mauchly’s tests and the Greenhouse-Geisser correction was applied when necessary. Shapiro-Wilk’s and Levene’s tests were used to test for normality in distributions and equality of variances.

## Results

**FIGURE 2:**
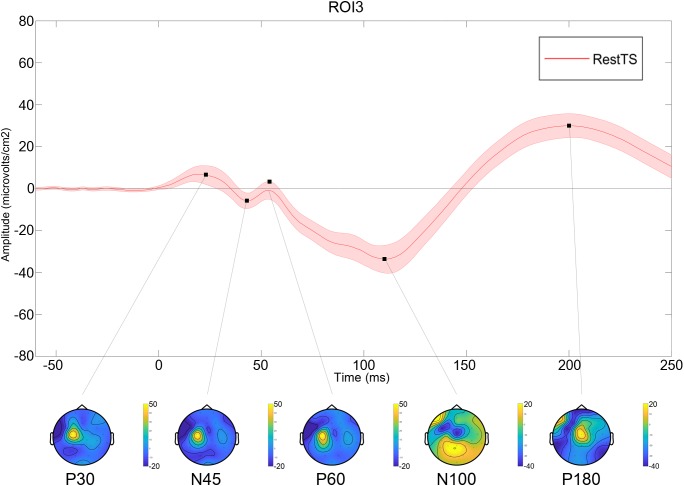
Averaged TEP at RestTS condition (*n* = 20). Upper panel: TEP in the centro-medial region (ROI3) (mean ± SE). Lower panel: topographic representation of current source density (CSD) for the main TEP peaks (yellow, positive values; blue, negative values).

All values are expressed as mean ± standard error. Twenty out of 24 subjects completed the experiment (mean age 42.2 ± 12.5; M/F: 12/8). Three subjects were excluded for having very high motor threshold and S50 greater than 100% maximal stimulator intensity. One subject was excluded due to poor compliance. The average number of trials included for analysis for a participant was: 79.7 ± 4.5 for RestTS, 75.8 ± 4.4 for RestPP, and 78.4 ± 5.5 for MovtTS. At RestTS, the TEP averaged across subjects and ROIs showed the typical components P30, N45, P60, N100, and P180 (Figure 2). P30, N45, and P60 showed a clear lateralization in the stimulated fronto-parietal region while N100 and P180 showed a widespread and bilateral distribution, with predominant centro-medial localization and extension, respectively to the left and right prefrontal regions.

In the following section, “reduction” reflects a change toward “less negative/more positive” values for negative peaks, and toward “less positive/more negative” values for positive peaks. Conversely, “increase” reflects a change toward “more negative/less positive” values for negative peaks, and toward “more positive/less negative” values for positive peaks.

### SICI

**FIGURE 3:**
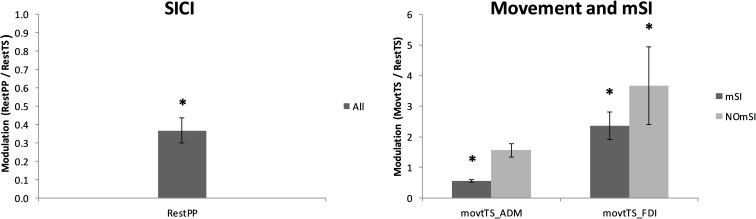
Averaged MEPs across conditions. Left: ADM MEP amplitude suppression by SICI (RestPP) is displayed as a ratio of 1 (RestTS). Right: ADM and FDI MEP amplitude modulation by movement onset (MovtTS) in subjects with and without motor surround inhibition (mSI) (mean ± SE; ^∗^*P* < 0.05).

Nineteen out of 20 participants (95%) showed a reduction of ADM MEP amplitude as a result of SICI paradigm. ADM MEP was significantly smaller in RestPP as compared with RestTS (1.94 ± 0.24 mV vs. 0.73 ± 0.13 mV, *t*(18) = 7.56, *p* < 0.001, a mean reduction of 1.22 (63%), 95% CI = 0.88, 1.55, *d* = 0.58) (Figure 3). One participant did not show SICI when comparing RestTS (0.82 ± 0.05 mV) and Rest PP (1.08 ± 0.05 mV).

The ANOVA that investigated the effect of paired pulses (SICI) on TEP amplitudes showed statistically significant three-way interaction ROI^∗^Condition^∗^Component. Further simple effects testing revealed significant two-way interactions – ROI^∗^Condition, ROI^∗^component and Condition^∗^Component, and a significant main effect of Component. Only ROI1 showed a statistically significant simple two-way interaction Condition ^∗^ Component. At ROI1 level, there was a statistically significant simple main effect of Condition for P30, N45, and P60 (Table 1).

**FIGURE 4:**
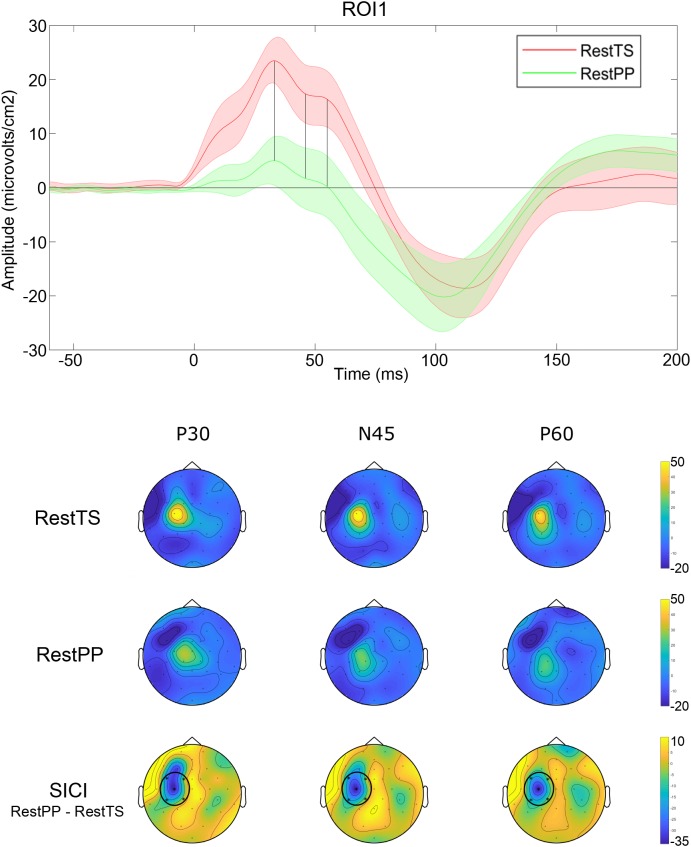
SICI related modulation of TEPs. Upper panel: Averaged TEP in ROI 1 at RestTS (red) and RestPP (green) (mean ± SE). P30, N45, and P60 were significantly modulated during SICI (vertical lines). Lower panel: topographic representation of current source density (CSD) for P30, N45, and P60 at RestTS (first row), RestPP (second row), and the effect of SICI (third row). ROI significantly modulated by SICI is highlighted (ROI1) (yellow, positive values; blue, negative values).

In summary, SICI was associated with reduced P30 and P60 and increased N45 at ROI1 level (Figure 4).

### Movement Onset – mSI

**FIGURE 5:**
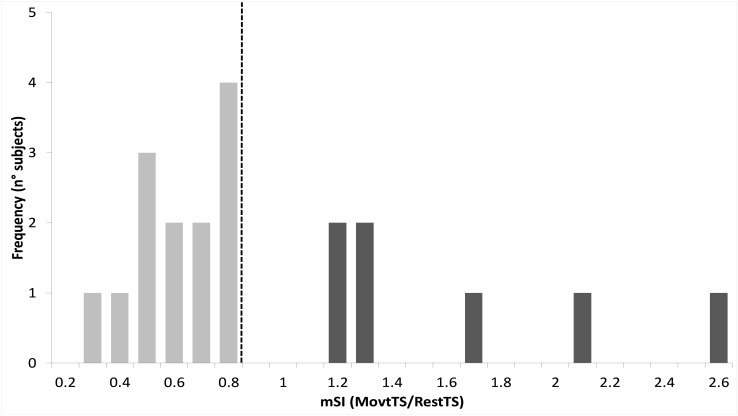
Histogram of motor surround inhibition (mSI) distribution. Vertical dashed line represents *a priori* cut-off value for mSI determination (movtTS/RestTS < 0.8). Subjects who showed lower values than the cut-off were assigned to mSI group (light gray), whereas those showing higher values were assigned as NOmSI group (dark gray).

Thirteen out of 20 participants (65%) showed mSI (mSI group) with a significant reduction of the ADM MEP in MovtTS as compared to RestTS [1.83 ± 0.22 mV vs. 1.02 ± 0.15 mV, *t*(12) = 5.43, *p* < 0.001, a reduction of 0.81 (44%), 95% CI 0.48, 1.13, *d* = 0.65]. The remaining seven subjects showed no mSI (NOmSI group) with a significant increase of the ADM MEP in MovtTS as compared to RestTS [2.00 ± 0.57 mV vs. 2.55 ± 0.48 mV, *t*(6) = -4.47, *p* = 0.004], an increase of 0.57 mV (29%), 95% CI = 0.26, 0.87, *d* = 0.58) (Figure 3). The histogram graph of the mSI distribution in our population showed a trend toward a bimodal distribution (Figure 5). There was no significant difference in ADM MEP amplitude at RestTS between the mSI and NOmSI groups [*t*(18) = -0.31, *p* = 0.76].

The mixed-design two-way ANOVA for the effect of movement on FDI MEP between mSI and NOmSI groups showed no significant Condition^∗^Group interaction [*F*(1,18) = 0.005, *p* = 0.95]. In all participants FDI MEP was significantly larger in MovtTS as compared to RestTS [2.87 ± 0.47 mV vs. 5.23 ± 0.42 mV, *t*(19) = 2.36, *p* < 0.001, an increase of 2.36 mV (82%), 95% CI = 1.73, 2.93, *d* = 0.57; Figure 3].

The four-way mixed ANOVA that investigated the effect of movement onset on TEPs amplitude showed statistically significant four-way interaction ROI^∗^Condition^∗^Component^∗^Group. Further simple effects testing revealed significant three-way interaction ROI^∗^Condition^∗^Component, Condition^∗^Component^∗^Group, a two-way interaction ROI^∗^Component and Condition^∗^Component, and a significant main effect of ROI and Component. A statistically significant simple two-way interaction Component^∗^Condition was found for all the ROIs: ROI1, ROI3, and ROI5. In ROI1, there was no statistically significant simple main effect of condition for any component. There was a statistically significant simple main effect of Condition for N100 in RO2, RO3, ROI4, and ROI5, and for P180 in ROI4. A significant three-way interaction ROI^∗^Condition^∗^Component was found only in Group mSI, and not in NOmSI group. When group mSI was considered, there was a statistically significant simple two-way interaction Condition^∗^Component in all the ROIs examined: ROI1, ROI2, ROI3, ROI4, and ROI5, and a statistically significant simple main effect of Condition was found in ROI1 for P30 and N45; for N100 in ROI3, ROI4, and ROI5; and for P180 in ROI4 (Table 2).

**Table 1:** SICI related modulation of TEPs amplitude – Three-way ANOVA: significant effects and interactions.

All subjects (*n* = 20)	df	*F*	*p*	η
**Interactions and main effects**				
ROI^∗^Condition^∗^Component	20, 380	1.90	0.011	0.091
ROI^∗^Condition	4, 76	5.23	0.012	0.216
ROI^∗^Component	20, 380	7.81	<0.001	0.29
Condition^∗^Component	5, 95	4.63	0.017	0.196
Component	5, 95	62.96	<0.001	0.77
**Simple interactions**				
Component^∗^Condition [ROI1]	5, 95	6.20	0.002	0.246
**Simple main effects**				
Condition [ROI1, p30]	1, 19	22.71	<0.001	0.544
Condition [ROI1, n45]	1, 19	12.55	0.002	0.398
Condition [ROI1, p60]]	1, 19	9.66	0.006	0.337

**Pairwise comparisons**	**RestTS (avg ± SE)**	**RestPP (avg ± SE)**	**Difference**	**95% CI**

p30, ROI1 (μV/cm^2^)	27.21 ± 4.64	8.98 ± 3.88	18.22	10.22, 26.23
n45, ROI1 (μV/cm^2^)	13.84 ± 4.20	−0.63 ± 4.76	14.47	5.92, 23.02
p60, ROI1 (μV/cm^2^)	17.83 ± 4.72	1.73 ± 4.91	16.10	5.26, 26.95

**FIGURE 6:**
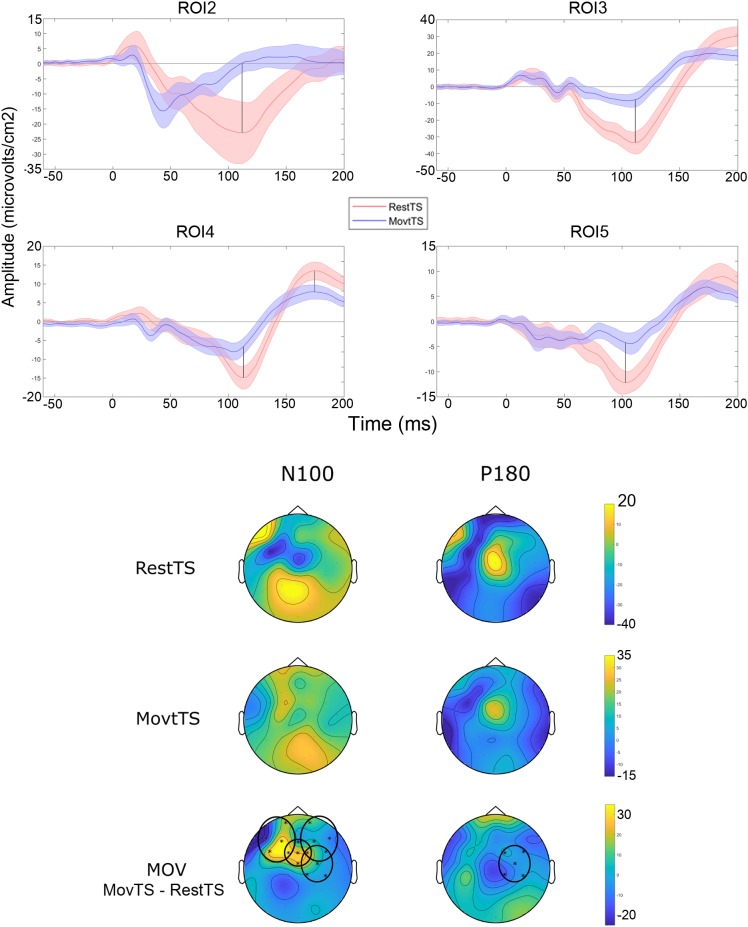
Movement related modulation of TEPs. Upper panel: Averaged TEP in ROIs that showed significant differences between RestTS (red) and MovtTS (blue) (mean ± SE). N100 was significantly modulated at movement onset in ROI2, 3, 4, and 5, while P180 was modulated in ROI4 (vertical lines). Lower panel: topographic representation of current source density (CSD) for N100 and P180 at RestTS (first row), MovtTS (second row), and the effect of movement onset (MOV, expressed as MovTS – RestTS) (third row). ROIs significantly modulated by MOV are highlighted (yellow, positive values; blue, negative values).

**FIGURE 7:**
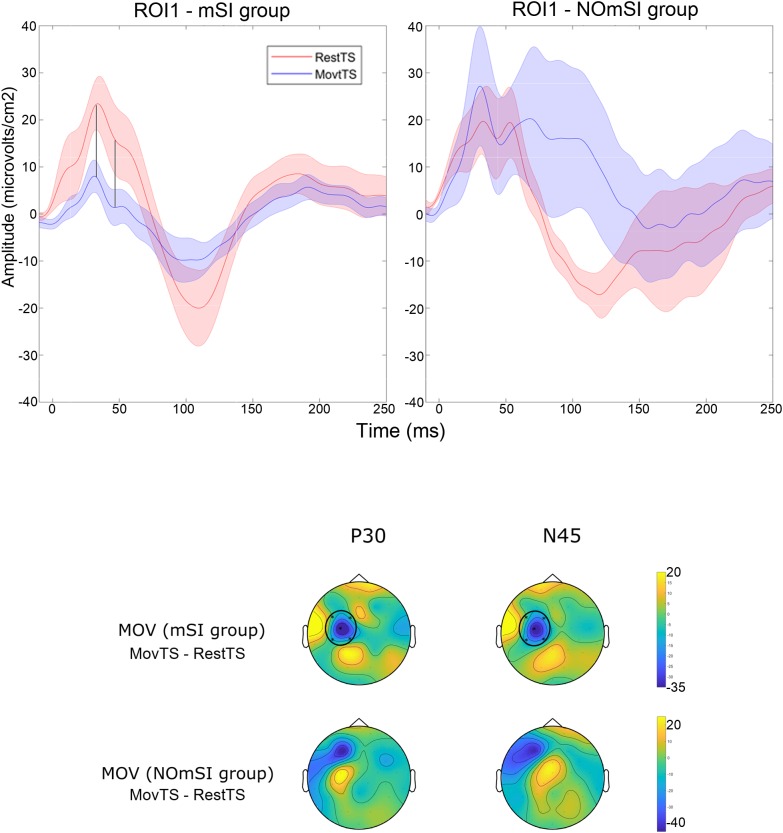
Motor surround inhibition related modulation of TEPs. Upper panel: Averaged TEP in ROI1 at RestTS (red), MovtTS (blue) in the mSI group (left), and in the NOmSI group (right) (mean ± SE). In ROI1, P30 and N45 were modulated at movement onset only in the mSI group (vertical lines). Lower panel: topographic representation of current source density (CSD) for the effect of movement onset (MOV, expressed as MovTS – RestTS) on P30 and N45 in the group with (first row), and without mSI (second row), ROIs significantly modulated by MOV are highlighted (yellow, positive values; blue, negative values).

In summary, at movement onset there was a reduction of N100 amplitude in ROI2, ROI3, ROI4 and ROI5, and of P180 amplitude in ROI4 (Figure 6). Movement onset affected TEPs in a different way in the mSI group as compared to the NOmSI group: only in the mSI group movement onset produced a reduction in P30 and N45 at ROI1 level (Figure 7).

## Discussion

In this TMS-EEG study on healthy subjects, SICI was associated with a reduction of P30 and P60, and an increase of N45 of TEPs recorded at the motor cortex. Movement onset was associated a widespread N100 reduction, and a more focal P180 reduction in the contralateral motor cortex. Only in people with mSI, movement onset reduced the P30 and increased the N45 at motor cortex level.

Somatosensory (SEP) and auditory evoked potentials (AEP) associated to TMS must be taken into account when interpreting TMS-EEG results (Conde et al., 2019). Since we did not use noise masking in our experiment, the AEP might have significantly contributed to N100 and P180 components (Nikouline et al., 1999; ter Braack et al., 2015). However, SICI and mSI modulated earlier TEP components within 60 ms, thus limiting a possible AEP effect. The influence of the SEP on TMS-EEG is yet to be demonstrated. A recent paper addressed the problem of sensory contamination in TMS-EEG and found no evidence of significant contamination before 60 ms after TMS pulse (Biabani et al., 2019). Since SICI and mSI modulated TEPs within 60 ms, we tend to exclude any relevant SEP related bias. Finally, supra-threshold TMS pulses produce reafferent components that may modulate P60 amplitude (Petrichella et al., 2017). Accordingly, SICI-associated P60 reduction might reflect a decreased proprioceptive feedback due to smaller MEPs. However, SICI proved to modulate TEPs elicited by dorsolateral prefrontal cortex stimulation at 60 ms suggesting this effect to be characteristic for SICI (Cash et al., 2017). Overall, evidence from previous works lead us to conclude that our results up to 60 ms are free of confounding factors related to the sensory stimulation associated with TMS, while greater caution should be used when interpreting the effects we found at 100 ms.

In the present paper, Laplacian TEPs showed temporal characteristics similar to non-Laplacian TEPs described in previous work (Komssi et al., 2004; Bonato et al., 2006; Premoli et al., 2014a; Cash et al., 2017; Petrichella et al., 2017). This result supports the validity of CSD estimates in TMS EEG studies and extends previous observation on other evoked potentials (Burle et al., 2015). In Laplacian TEPs, positive values represent current flow from the cortex to the scalp, and negative values represent current flow from the scalp into the cortex (Nunez and Srinivasan, 2006). Therefore, change in TEPs amplitude likely reflects a modulation of the underpinning brain activity. By demonstrating that SICI modulated TEPs up to 60 ms, our study supports the relation between early TEP components and local cortical excitability and confirms the findings of past studies (Ferreri et al., 2011; Cash et al., 2017). GABA-Ar mediated inhibitory activity may contribute to early recordable TEP components (Connors et al., 1988; Davies et al., 1990; Kaila et al., 1993; Deisz, 1999). Supporting an association between SICI and GABA-Ar activity, SICI increased N45 amplitude, a component linked to GABA-Aergic tone (Premoli et al., 2014a). However, a more recent study found no effect of SICI on the early TEPs (Premoli et al., 2018). Premoli et al. (2018) used different stimulation intensities for both the CS (70% RMT in their study vs. 80% in ours), and more relevant for the TS (100% RMT in their study vs. S50 in ours). Our TS was about 120%RMT and always elicited a MEP (larger than 1 mV on average), which guarantees a consistent activation of the cortico-spinal pathways. The discrepancy in SICI effect on TEPs may lie in the different amount of cortical activation produced by the two different TS intensities. Finally, what also differs from Premoli et al. is that we did not subtract CS-alone TEP from the paired-pulse TEP. According to Premoli et al. the CS alone produces a TEP (CS TEP) similar to that of the TS (TS TEP). Since the TEP is a sequence of time-locked 20–35 Hz oscillations occurring in the first 100 ms, followed by 8 to 12 Hz oscillations that persist until 300 ms, the expected effect of the arithmetic summation of the CS and the TS TEPs, with a 2 ms ISI (i.e., 500 Hz – an order of magnitude faster), is a non-specific increase of almost in-phase components (Ilmoniemi and Kicić, 2010). On the contrary, we observed a decrease in amplitude of the early positive components and an increase only for the N45. Thus, we believe that SICI-associated effect on early TEPs cannot be explained summation of CS and TS TEPs.

The result that N100 is decreased at the beginning of the movement is a novel finding and is in line with previous work that found a reduction of N100 during movement preparation (Nikulin et al., 2003; Bender et al., 2005; Kičić et al., 2008). N100 has been linked to GABA type B receptor (GABA-Br) mediated inhibition (Premoli et al., 2014a,b). The widespread reduction of N100 that we observed at movement onset might be the result of a reduced level of cortical GABA-Br inhibitory activity during movement execution. The P180 modulation at movement onset seemed to follow N100 trend although limited to the fronto-central ROI contralateral to the stimulated side. One study showed that P180 is modulated similar to N100 following TMS paradigms associated with GABA-Br mediated cortical inhibition (Premoli et al., 2014b). However, little is known about the mechanism underlying the P180 component, and the AEP and the SEP may contribute significantly to its amplitude thus limiting any conclusion regarding this component (Gordon et al., 2018).

**Table 2:** Movement onset and mSI related modulation of TEPs amplitude – Four-way ANOVA: significant effects and interactions.

All subjects (*n* = 20)	df	*F*	*P*	η
**Interactions and main effects**				
ROI^∗^Condition^∗^Component^∗^Group	20, 360	1.91	0.011	0.096
ROI^∗^Condition^∗^Component	20, 360	3.35	<0.001	0.157
Condition^∗^Component^∗^Group	5, 90	2.38	0.044	0.117
ROI^∗^Component	20, 360	9.17	<0.001	0.338
Condition^∗^Component	5, 90	21.44	<0.001	0.544
ROI	4, 72	7.18	<0.001	0.285
Component	5, 90	69.87	<0.001	0.795
**Simple interactions**				
Component^∗^Condition [ROI1]	5, 95	5.02	0.005	0.209
Component^∗^Condition [ROI2]	5, 95	3.74	0.042	0.164
Component^∗^Condition [ROI3]	5, 95	17.93	<0.001	0.486
Component^∗^Condition [ROI4]	5, 95	7.91	0.001	0.294
Component^∗^Condition [ROI5]	5, 95	6.04	<0.001	0.241
**Simple main effects**				
Condition [ROI2, n100]	1, 19	5.36	0.032	0.220
Condition [ROI3, n100]	1, 19	33.40	<0.001	0.637
Condition [ROI4, n100]	1, 19	14.69	0.001	0.436
Condition [ROI4, n100]	1, 19	13.84	0.001	0.421
Condition [ROI4, p180]	1, 19	11.31	0.003	0.373

**Pairwise comparisons**	**RestTS (avg ± SE)**	**MovtTS (avg ± SE)**	**Difference**	**95% CI**

n100, ROI2 (μV/cm^2^)	−30.64 ± 9.72	−10.33 ± 3.36	−20.38	−38.81, −1.095
n100, ROI3 (μV/cm^2^)	−42.69 ± 6.09	−12.81 ± 3.42	−29.88	−40.70, −19.06
n100, ROI4 (μV/cm^2^)	−18.11 ± 2.69	−10.83 ± 2.12	−7.28	−11.26, −3.31
n100, ROI5 (μV/cm^2^)	−17.01 ± 2.43	−8.01 ± 2.01	−9.06	−14.16, −3.96
p180, ROI4 (μV/cm^2^)	17.32 ± 1.69	10.32 ± 1.65	7.00	2.64, 11.36

**Group mSI (*n* = 13)**	**df**	***F***	***p***	**η**

**Interactions**				
ROI^∗^Condition^∗^Component	20, 240	3.91	<0.001	0.246
**Simple interactions**				
Component^∗^Condition [ROI1]	5, 60	4.56	0.026	0.275
Component^∗^Condition [ROI2]	5, 60	3.58	0.007	0.230
Component^∗^Condition [ROI3]	5, 60	10.41	< 0.001	0.465
Component^∗^Condition [ROI4]	5, 60	3.71	0.005	0.236
Component^∗^Condition [ROI5]	5, 60	5.74	<0.001	0.324
**Simple main effects**				
Condition [ROI1, p30]	1, 12	11.70	0.005	0.494
Condition [ROI1, n45]	1, 12	9.03	0.011	0.429
Condition [ROI3, n100]	1, 12	25.47	<0.001	0.680
Condition [ROI4, n100]	1, 12	8.07	0.015	0.402
Condition [ROI4, p180]	1, 12	5.38	0.039	0.310

**Pairwise comparisons**	**RestTS (avg ± SE)**	**MovtTS (avg ± SE)**	**Difference**	**95% CI**

p30, ROI1 (μV/cm^2^)	28.58 ± 6,92	8.57 ± 3.77	20.00	7.27, 32.74
n45, ROI1 (μV/cm^2^)	10.69 ± 6.24	−0.12 ± 3.58	10.81	2.97, 18.65
n100, ROI3 (μV/cm^2^)	−36.41 ± 7.08	−10.30 ± 5.07	−26.11	−37.38, −14.84
n100, ROI4 (μV/cm^2^)	−16.47 ± 3.38	−10.55 ± 2.94	−5.9	−10.46, −1.38
n100, ROI5 (μV/cm^2^)	−17.41 ± 2.95	−6.91 ± 1.94	−10.50	−16.03, −4.96
p180, ROI4 (μV/cm^2^)	17.20 ± 2.22	10.63 ± 2.35	6.54	0.40, 12.75
**Group NOmSI (*n* = 7)**				

**Interactions**	**df**	***F***	***p***	**η**

ROI^∗^Condition^∗^Component	20, 120	1.51	0.089	*–*

We have now found that mSI modulated P30 and N45 amplitudes within motor cortex. This is the first work that identifies cortical correlates of mSI. The observation that in the NOmSI group we did not see any modulation of P30 or N45 components excludes non-specific effects due to movement onset and strengthens the conclusion that these are mSI specific correlates. The TEP is determined by both the characteristics of the stimulus and the state of the stimulated cortical circuit (Casarotto et al., 2010). Delivering TMS on the “surround” muscle’s cortical representation, and applying a Laplacian filter known for dramatically reducing cortical volume conduction (Burle et al., 2015) could be the reason why our experimental approach succeeded in isolating the cortical correlate of a localized phenomenon such as mSI. The observation that some participants failed to show mSI, is in line with previous observations (Sadnicka et al., 2013). Moreover, it is known that mSI is an adaptable phenomenon subject to plasticity (Kassavetis et al., 2012; Belvisi et al., 2014). Subtle differences in the mode of execution of the motor task, or somatotopic organization within motor cortex may have been sufficient for some subjects to not show mSI for the explored pair of muscles. Finally, the histogram of mSI values showed a trend towards a bimodal distribution, although the small sample size limits the validity of this conclusion. This result, together with the observation that no participant showed mSI values in the range between 0.8 and 1.2, suggests that the group assignment reflected a true dichotomous inhibition/facilitation phenomenon and not simply a median split on normally distributed data.

Previous works found conflicting results investigating SICI contributions to the genesis of mSI. Stinear and Byblow found SICI from a surround muscle to be enhanced during movement, while Beck et al. (2008) could not replicate the same result (Stinear and Byblow, 2003). Our results showed that SICI and mSI were associated with very similar effects thus supporting the hypothesis that local intracortical inhibition, as tested by SICI, contributes to the genesis of mSI. Within the motor cortex, pyramidal neurons send excitatory horizontal collaterals to other pyramidal neurons, as well as to inhibitory interneurons (Hendry and Jones, 1981; Keller, 1993). The connections between pyramidal neurons and the inhibitory interneurons may constitute the substrate for the mSI while the inhibitory interneurons may be the final effector of both SICI and mSI. Supporting this hypothesis, both SICI and mSI modulated N45, a TEP component that has been found to be related to GABA-A-ergic tone (Premoli et al., 2014a).

We acknowledge a technical limit to our study. It is important to consider that cortical activity directly related to MEP takes place within the first 5 ms after the TMS pulse (Esser et al., 2005; Li et al., 2017). This initial brief excitatory phase is too short for any of our TEP components to directly reflects the effects of SICI and mSI on MEP-associated cortical activity. Yet, TEPs later than 5 ms are indirectly informative of the functional state of the stimulated cortex since the inhibitory phases that follow the excitatory one last long enough to be recorded (Connors et al., 1988; Deisz, 1999; Ferreri et al., 2011).

In conclusion, SICI and mSI are associated with similar modulations of TEPs. Therefore, the current TMS-EEG study has added new knowledge about the mechanisms involved in motor cortical inhibition. The cortical activity induced by a conditioning stimulus for SICI and the cortical activity occurring at movement onset in a surround area share similar mechanisms. Our results can be used to develop novel study hypotheses aimed at exploring the mechanisms underlying abnormal cortical inhibition in movement disorders such as Parkinson’s disease and focal hand dystonia.

## Data Availability

The datasets generated for this study are available on request to the corresponding author.

## Ethics Statement

This study was carried out in accordance with the recommendations of Declaration of Helsinki with written informed consent from all subjects. The protocol was approved by the Neuroscience Institutional Review Board of the National Institutes of Health.

## Author Contributions

All authors contributed to the design, interpretation of the results, critically revised the manuscript, and provided the final approval of the version to be published. GL, NT, and HC collected and analyzed the data. GL and NT drafted the manuscript.

## Conflict of Interest Statement

MH serves as Chair of the Medical Advisory Board for and may receive honoraria and funding for travel from the Neurotoxin Institute. He may accrue revenue on US Patent #6,780,413 B2 (Issued: August 24, 2004): Immunotoxin (MAB-Ricin) for the treatment of focal movement disorders, and US Patent #7,407,478 (Issued: August 5, 2008): Coil for Magnetic Stimulation and methods for using the same (H-coil); in relation to the latter, he has received license fee payments from the NIH (from Brainsway) for licensing of this patent. He is on the Medical Advisory Boards of CALA Health and Brainsway. He is on the Editorial Board of approximately 15 journals, and received royalties and/or honoraria from publishing from Cambridge University Press, Oxford University Press, and Elsevier. MH’s research at the NIH was largely supported by the NIH Intramural Program. Supplemental research funds have been granted by Merz for treatment studies of focal hand dystonia, Allergan for studies of methods to inject botulinum toxins, Medtronic, Inc., for a study of DBS for dystonia, and CALA Health for studies of a device to suppress tremor. The remaining authors declare that the research was conducted in the absence of any commercial or financial relationships that could be construed as a potential conflict of interest. The handling Editor declared a shared affiliation, though no other collaboration, with one of the authors AB at the time of review.
